# Machine Learning Integration with Single-Cell Transcriptome Sequencing Datasets Reveals the Impact of Tumor-Associated Neutrophils on the Immune Microenvironment and Immunotherapy Outcomes in Gastric Cancer

**DOI:** 10.3390/ijms252312715

**Published:** 2024-11-26

**Authors:** Jingcheng Zhang, Mingsi Zhang, Jiaheng Lou, Linyue Wu, Shuo Zhang, Xiaojuan Liu, Yani Ke, Sicheng Zhao, Zhiyuan Song, Xing Bai, Yan Cai, Tao Jiang, Guangji Zhang

**Affiliations:** 1 School of Basic Medical Sciences, Zhejiang Chinese Medical University, Hangzhou 310053, Chinaljh990704@163.com (J.L.); wulinyue1999@163.com (L.W.); zs15156020951@163.com (S.Z.); 15832362719@163.com (X.L.); 201512201503019@zcmu.edu.cn (Y.K.); xczyzsc@163.com (S.Z.); 16639178019@163.com (Z.S.); baixing0900@163.com (X.B.); chaeyeon97@163.com (Y.C.); 2 Zhejiang Key Laboratory of Blood-Stasis-Toxin Syndrome, Zhejiang Chinese Medical University, Hangzhou 310053, China; 3 Musculoskeletal Sport Science and Health, Loughborough University, Loughborough LE11 3TU, UK; m.zhang4-23@student.lboro.ac.uk

**Keywords:** gastric cancer, neutrophils, immunotherapy, single-cell sequencing, machine learning

## Abstract

The characteristics of neutrophils play a crucial role in defining the tumor inflammatory environment. However, the function of tumor-associated neutrophils (TANs) in tumor immunity and their response to immune checkpoint inhibitors (ICIs) remains incompletely understood. By analyzing single-cell RNA sequencing data from over 600,000 cells in gastric cancer (GSE163558 and GSE183904), colorectal cancer (GSE205506), and lung cancer (GSE207422), we identified neutrophil subsets in primary gastric cancer that are associated with the treatment response to ICIs. Specifically, we focused on neutrophils with high expression of *CD44* (CD44_NEU), which are abundant during tumor progression and exert significant influence on the gastric cancer immune microenvironment. Machine learning analysis revealed 22 core genes associated with CD44_NEU, impacting inflammation, proliferation, migration, and oxidative stress. In addition, multiple immunofluorescence staining and gastric cancer spatial transcriptome data (GSE203612) showed a correlation between CD44_NEU and T-cell infiltration in gastric cancer tissues. A risk score model derived from seven essential genes (*AQP9*, *BASP1*, *BCL2A1*, *PLEK*, *PDE4B*, *PROK2*, and *ACSL1*) showed better predictive capability for patient survival compared to clinical features alone, and integrating these scores with clinical variables resulted in a prognostic nomogram. Overall, this study highlights the heterogeneity of TANs, particularly the CD44_NEU critical influence on immunotherapy outcomes, paving the way for personalized treatment strategies.

## 1. Introduction

Neutrophils, the sentinels of immunity, manifest a dual-edged sword within the tumor microenvironment [1,2,3]. They orchestrate anti-tumor immunity by deploying pro-inflammatory factors and reactive oxygen species, yet paradoxically, they can also unleash matrix metalloproteinases (MMPs) and other factors that foster tumor invasion and metastasis [4,5]. Recent research advancements have unveiled intricate molecular mechanisms underlying neutrophil “betrayal”, including activation by tumor cells via various signaling pathways, such as the *NAMPT-NTRK1* signaling axis, and the expression of immunosuppressive cytokines like *IL-10* and *TGF-β* [6,7]. This creation of an immunosuppressive microenvironment significantly undermines immunotherapy response and portends a grim prognosis, underscoring the critical role of neutrophils in shaping the outcome of selected tumor patients.

Immune checkpoint inhibitors (ICIs), a seminal breakthrough in cancer immunotherapy, empower the immune system to renew its assault on tumors by disrupting immunosuppressive signaling pathways, thereby executing an antitumor function. In clinical settings, drugs such as nivolumab and atezolizumab have demonstrated therapeutic efficacy in select patients, enhancing survival rates and boosting treatment response [8,9,10]. Although ICIs has shown some efficacy in gastric cancer (GC) patients, there are still some patients who are not sensitive to ICIs treatment [11]. The effectiveness of ICIs is contingent upon various factors, including pathological traits, immune cell composition, and cytokine profiles. Contemporary research underscores the likelihood that the varying efficacy of ICIs is intimately tied to the diversity and functional dynamics of immune cells within the tumor microenvironment, particularly the intricate multifaceted role played by neutrophils [12].

The current scarcity of predictive biomarkers for ICIs’ efficacy and the limited combination therapies hinder the widespread and optimal clinical utilization of these agents [13]. To elucidate the precise role of neutrophils in ICI therapy and its underlying molecular mechanisms, we require an advanced technology capable of dissecting the intricate heterogeneity of the tumor microenvironment. Single-cell RNA sequencing (scRNA-seq), a leap from traditional RNA sequencing, offers a holistic view of gene expression patterns while introducing a novel dimension to tumor biology exploration—cell-level heterogeneity. By scrutinizing gene expression at the single-cell resolution, scRNA-seq unmasks hitherto undiscovered cellular diversity and heterogeneity within the tumor niche. In the context of GC, both human tissue and mouse models have leveraged scRNA-seq to stratify tumor-associated neutrophils (TANs) into distinct subsets, elucidating the nuanced biological roles of these subgroups [14]. This approach promises to facilitate the discovery of novel biomarkers and illuminate the specific response patterns of cell subsets to immunotherapy, thereby paving the way for tailored therapeutic strategies [15,16].

Given the aforementioned backdrop, this study endeavors to delve into the alterations in crucial gene expression profiles of tumor-associated neutrophils (TANs) using the scRNA-seq database in conjunction with transcriptomic data and an ICI treatment cohort. Our objective is to meticulously examine the relationship between these transcriptional changes and ICI efficacy. By so doing, we aspire to uncover the potential mechanisms underlying TANs’ role in ICI therapy, establish predictive models grounded in key gene signatures, and ultimately contribute to more tailored and precise treatment recommendations that inform clinical decision making.

## 2. Results

**Figure 1:**
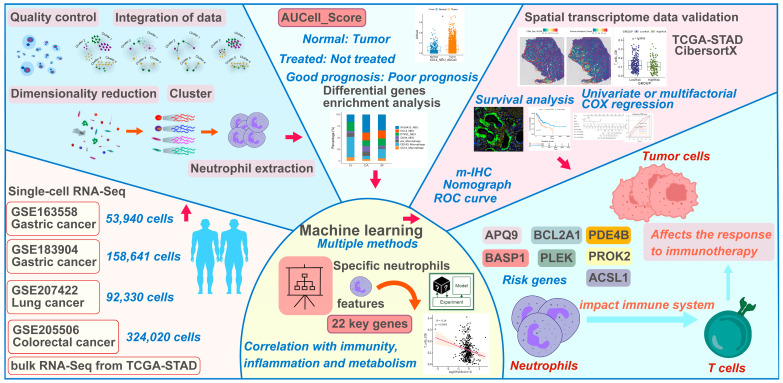
Schematic diagram of the entire research process.

The overall study design is illustrated in Figure 1.

### 2.1. Single-Cell Sequence Analysis and Cell Type Identification

**Figure 2:**
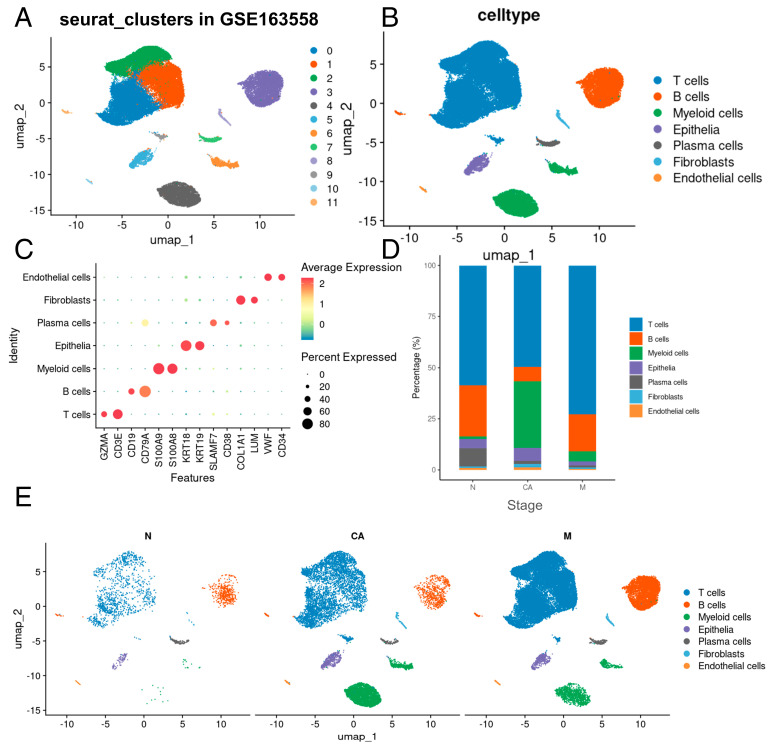
Identification of infiltrating cell types. (**A**,**B**) Single-cell plots colored by cell type in GSE163558. (**C**) Markers used for cell identification. (**D**) Relative proportions of cell types. The relative contribution of each population is weighted by cell number and scaled to 100%. (**E**) Overall landscape of single cells at different stages. N, normal; CA, cancer; M, metastasis.

Upon implementing rigorous quality control measures and eliminating batch effects, 53,940 single cells from the GSE163558 scRNA-seq dataset, encompassing normal tissue, GC tissue, and GC metastatic tissue, were systematically clustered into 12 distinct main clusters (Figure 2A). These clusters were annotated with cell type-specific genes utilizing established classical markers (Figure 2B,C).

To delve into the intricacies of tumor microenvironment (TME) remodeling, we quantified the proportional representation of various cell types across samples (Figure 2D). Additionally, Figure 2E highlights the notable variations in cellular composition among samples at different disease stages. Notably, significant changes were observed in myeloid cells, with a significant increase in the proportion of myeloid cells in tumor and metastatic tumor samples.

### 2.2. Identification and Validation of Tumor-Associated Neutrophils in GC

**Figure 3:**
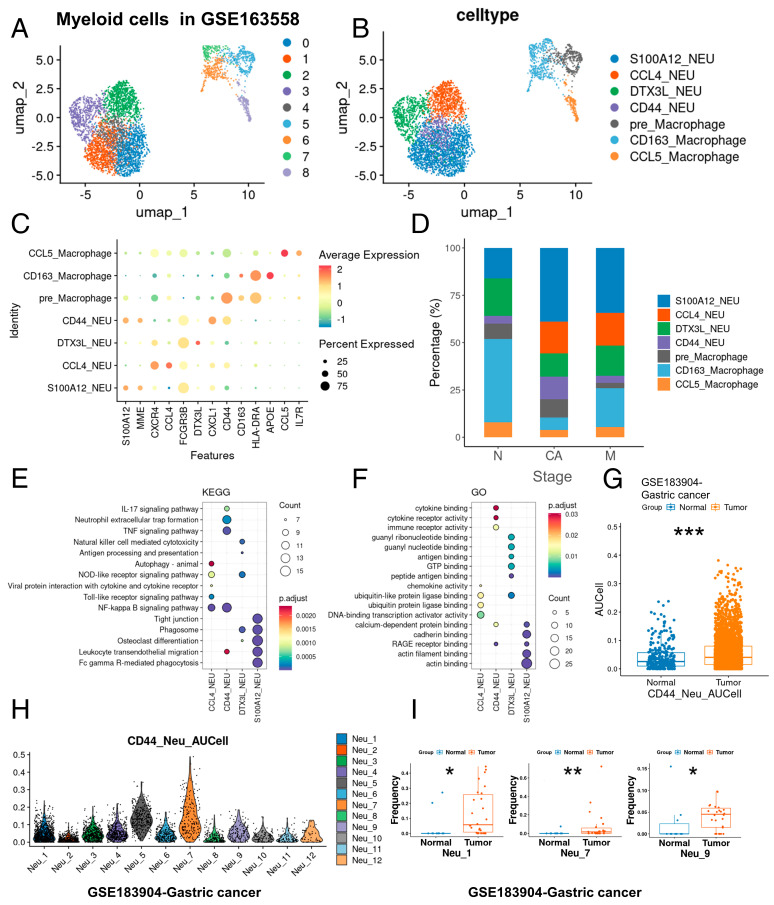
Identification of tumor-associated neutrophils. (**A**,**B**) Myeloid cells plots colored by cell type in this study. (**C**) MARKERS used for cell identification. (**D**) Relative proportions of myeloid cells types. The relative contribution of each population is weighted by cell number and scaled to 100%. N, normal; CA, cancer; M, metastasis. (**E**) Top five functional enrichment results of neutrophil subpopulations in the GSE163558 dataset from the KEGG database. (**F**) Top five functional enrichment results of neutrophil subpopulations in the GSE163558 dataset from the GO database. NEU, neutrophil. (**G**) Comparison of CD44_NEU feature-related AUCell scores between normal and tumor tissues in the GSE183904 dataset. (**H**) CD44_NEU feature-related AUCell scores of neutrophil subpopulations in the GSE183904 dataset. (**I**) Comparison of Neu_1, Neu_7, and Neu_9 relative proportions between normal and tumor tissues in the GSE183904 dataset. * < 0.05; ** < 0.01; *** < 0.001.

In order to further clarify the role of myeloid cells, we extracted myeloid cells to reduce dimension again into nine subgroups (Figure 3A) and identified four neutrophil subgroups and three macrophage subgroups by cluster specific genes (Figure 3B,C). We observed that the proportion of certain neutrophil subsets increased specifically during the tumor and metastatic stages (Figure 3D), with a notable increase in neutrophils exhibiting high expression of *CD44* (CD44_NEU), specifically at the tumor stage. Concurrently, neutrophils exhibiting high expression of *CCL4* (CCL4_NEU) were specifically present during both tumor and metastatic stages. Subsequently, we used the KEGG and GO databases to perform enrichment analysis on the differential genes identified among the neutrophil populations calculated using the FINDALLMAREKR function (PCT > 0.3, P_adj < 0.05, Supplementary Material S1). Our analysis revealed that CD44_NEU was significantly enriched in the IL-17 signaling pathway, neutrophil extracellular trap formation, TNF signaling pathway, NF-kappa B signaling pathway, leukocyte transendothelial migration, cytokine binding, cytokine receptor activity, immune receptor activity, and other pathways. Meanwhile, CCL4_NEU was involved in autophagy, NOD-like receptor signaling pathway, viral protein-cytokine interaction, toll-like receptor signaling pathway, NF-kappa B signaling pathway, chemokine activity, ubiquitin-like protein ligase binding, ubiquitin-protein ligase binding, and DNA-binding transcriptional activator activity, among others (Figure 3E,F). This suggests that CD44_NEU and CCL4_NEU may participate in the remodeling of the tumor immune microenvironment.

We then utilized the GSE183904 GC dataset to further validate our previous findings. Using a similar approach, we extracted neutrophils from the GSE183904 dataset and reclustered them into 12 neutrophil subgroups (Figure S1A–E). AUCell scores are a method used to assess the activity level of a specific gene set in a single cell or cell population in the analysis of single-cell RNA sequencing data. This method evaluates the activity of a gene set by calculating the expression rank of genes within the gene set in the cell. Initially, we employed the AUCell package to calculate AUCell scores for neutrophils in both normal and tumor samples, using the differentially expressed genes associated with CD44_NEU and CCL4_NEU subsets as scoring genes. We found that both scores were significantly elevated during the tumor stage (Figure 3G and Figure S1H). Furthermore, we calculated the CD44_NEU feature-related AUCell scores and CCL4_NEU feature-related AUCell scores for the 12 neutrophil subsets and identified neutrophil subsets similar to CD44_NEU and CCL4_NEU in GSE183904 (Figure 3H and Figure S1G). We noticed that neutrophil subsets such as Neu_1, Neu_4, Neu_5, Neu_6, Neu_7, and Neu_9 all had high CD44_NEU feature-related AUCell scores, and the proportions of Neu_1, Neu_7, and Neu_9 increased significantly in the tumor stage (Figure 3I and Figure S1F). This suggests that CD44_NEU content is indeed specifically elevated at the tumor stage.

### 2.3. Machine Learning-Based Core Gene Mining and Functional Identification of CD44_NEU

**Figure 4:**
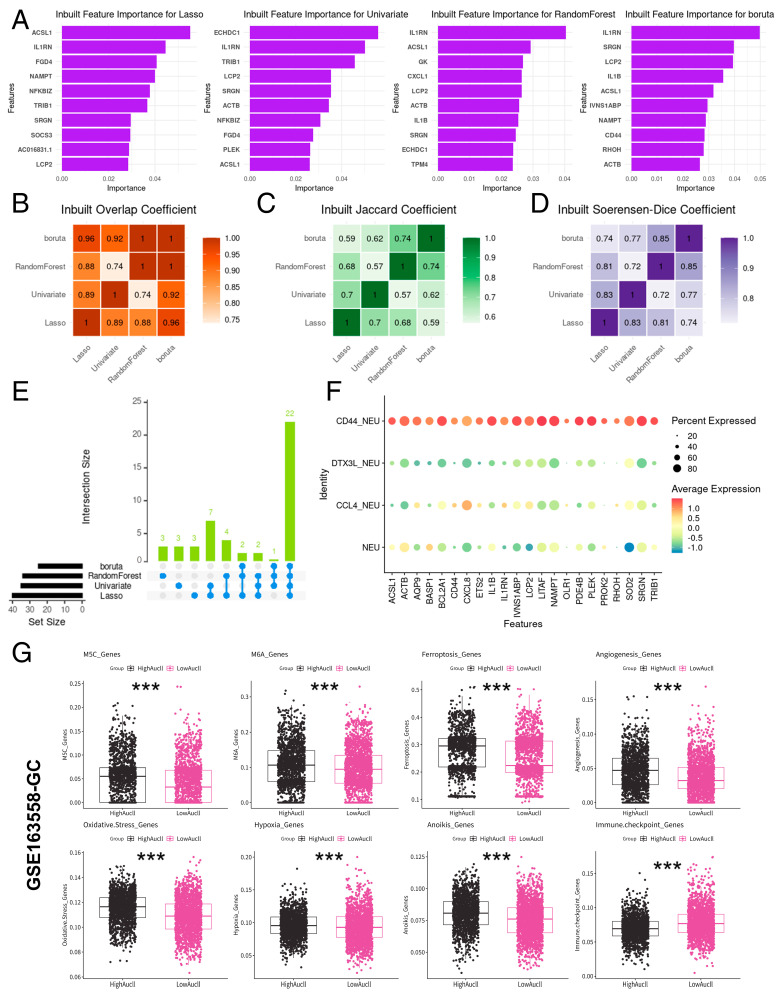
Identification and functional analysis of CD44_NEU core gene characteristics. (**A**) Top 10 genes in Lasso, Univariate, RandomForest, and boruta machine learning results. (**B**–**D**) Comparison of overlap coefficient, Jaccard coefficient, and Soerensen–Dice coefficient in Lasso, Univariate, RandomForest, and boruta machine learning results. (**E**) A Venn diagram was created to visualize the gene intersections obtained from four different machine learning algorithms, using the R for the visualization. (**F**) Expression of 22 key functional genes in neutrophil subpopulations in the GSE163558 dataset. (**G**) AUCell scores of eight functional gene sets were compared after grouping according to the mean of CD44_NEU feature-related AUCell scores in the GSE163558 dataset. *** < 0.001.

Our study focused on the relationship between the specific neutrophils and the outcome of ICIs treatment, and the specific rise of CD44_NEU at the tumor stage is related to the activity of immune receptors and other biological processes in situ tumors, so we conducted further studies on the characteristics of CD44_NEU To further explore its function; we used the Lasso, Univariate, RandomForest, and boruta machine learning algorithms in the GeneSelectR package to identify core genes in CD44_NEU. Figure 4A shows the top 10 genes sorted by weight of the four algorithms, and Figure 4B–D show the overlap coefficient, Jaccard coefficient, and Soerensen–Dice coefficient of the four algorithms. Subsequently, we identified the overlapping genes of the four methods (Figure 4E) and finally obtained 22 genes strongly related to the characteristics of CD44_NEU (Figure 4F, Supplementary Material S2).

**Figure 5:**
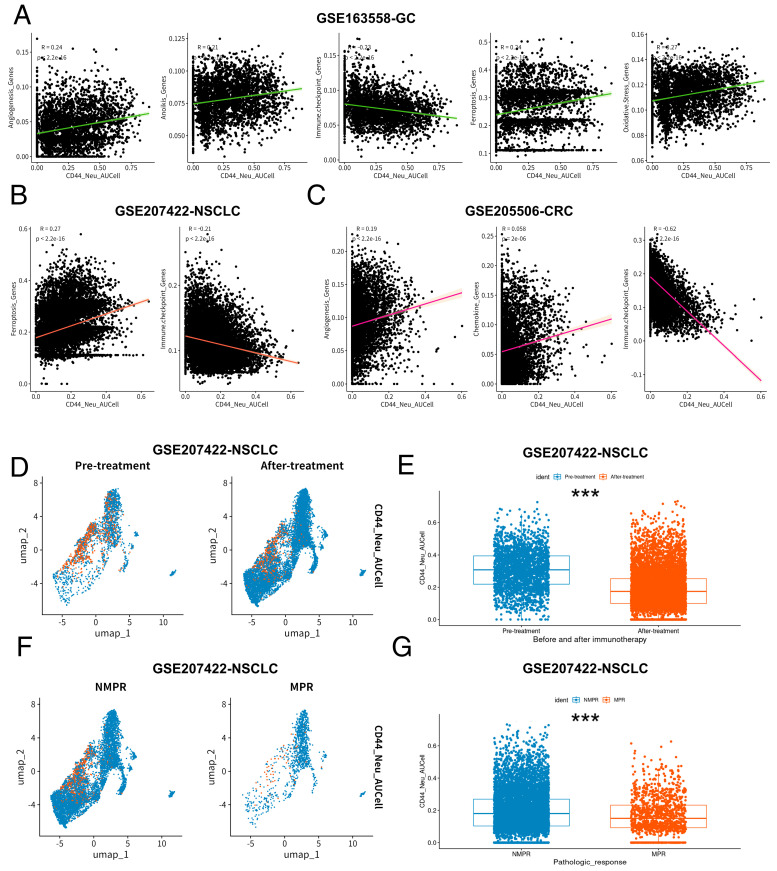
Functional analysis and correlation with immune efficacy of CD44_NEU core gene characteristics. (**A**) Correlation of CD44_NEU feature-related AUCell scores with angiogenesis genes (*r* = 0.24, *p* < 0.05), anoikis genes (*r* = 0.21, *p* < 0.05), immune checkpoint genes (*r* = 0.23, *p* < 0.05), ferroptosis genes (*r* = 0.24, *p* < 0.05), oxidative stress genes (*r* = 0.27, *p* < 0.05), and in GSE163558. GC, gastric cancer. (**B**) Correlation of CD44_NEU feature-related AUCell scores with ferroptosis genes (*r* = 0.27, *p* < 0.05) and immune checkpoint genes (*r* = 0.21, *p* < 0.05) in GSE207422. NSCLC, non-small-cell lung cancer. (**C**) Correlation of CD44_NEU feature-related AUCell scores with angiogenesis genes (*r* = 0.19, *p* < 0.05), chemokine genes (r = 0.058, *p* < 0.05), and immune checkpoint genes (*r* = −0.62, *p* < 0.05) in GSE205506. CRC, colorectal cancer. (**D**,**E**) UMAP plots and CD44_NEU feature-related AUCell scores comparisons of neutrophils before and after immunotherapy in GSE207422 (orange represents high AUCell score; blue represents low AUCell score; the grouping is based on CD44_NEU feature-related AUCell scores). (**F**,**G**) UMAP plots and CD44_NEU feature-related AUCell scores comparisons of neutrophils in NMPR (non-major pathological remission) and MPR (major pathological remission) samples in GSE207422 (orange represents high AUCell score; blue represents low AUCell score; the grouping is based on the average of CD44_NEU feature-related AUCell scores). *** < 0.001.

We summarized 11 functional gene sets based on previous studies (angiogenesis genes, anoikis genes, autophagy-related genes, chemokine genes, cuproptosis genes, ferroptosis genes, hypoxia genes, immune checkpoint genes, M5C genes, M6A genes, and oxidative stress genes) performed using the same AUCell scores assessment on neutrophils (Supplementary Material S3). To evaluate the specific biological functions to which neutrophils are related, we calculated 11 functional gene sets from the AUCell scores of neutrophils in the GSE163558 dataset using AUcell and compared the score differences among the four groups of neutrophil subpopulations (Figure S2A–K). Subsequently, the CD44_NEU feature-related AUCell scores of each neutrophil in the GSE163558 dataset were recalculated based on the core gene set calculated by machine learning. Then, based on the average value of the CD44_NEU feature-related AUCell scores, cells were divided into a high-AUCell group and low-AUCell group. The results showed that the AUCell scores of M5C features, M6A features, ferroptosis features, angiogenesis features, oxidative stress features, and anoikis features in the high-AUCell group were significantly increased, and the score of immune checkpoint features was significantly decreased (*p* < 0.05, Figure 4G). In addition, in the GSE165338 dataset, CD44_NEU feature-related AUCell scores showed a weak positive correlation with angiogenesis characteristics, anoikis characteristics, ferroptosis characteristics, and oxidative stress characteristics and a weak negative correlation with immune checkpoint characteristics (*p* < 0.05, Figure 5A). In the field of biology, although the microenvironments of different tumors exhibit significant variability, there is also a noticeable similarity between them [17,18,19,20]. This similarity provides us with a unique perspective to explore the immune microenvironment of specific tumors, such as gastric cancer, through cross-cancer analysis. Therefore, we adopted a multi-cancer dataset for comprehensive analysis. To understand whether core gene sets have biological significance in more cancers, we used the same method for neutrophils in the GSE207422 dataset and found a weak positive correlation between CD44_NEU feature-related AUCell scores and ferroptosis characteristics. There still was a weak negative correlation with immune checkpoint feature scores (*p* < 0.05, Figure 5B). Further, in GSE205506, we found that the CD44_NEU feature-related AUCell scores of its neutrophils also had a weak positive correlation with angiogenesis characteristics and chemokine characteristics and a moderate negative correlation with immune checkpoint characteristics (*p* < 0.05, Figure 5C). The above results prove that CD44_NEU feature-related AUCell scores are indeed correlated with various cellular processes, such as immunity and apoptosis.

### 2.4. CD44_NEU Core Genes Are Related to Tumor Immunotherapy, Tumor Immunotherapy Efficacy, and Tumor Prognosis

**Figure 6:**
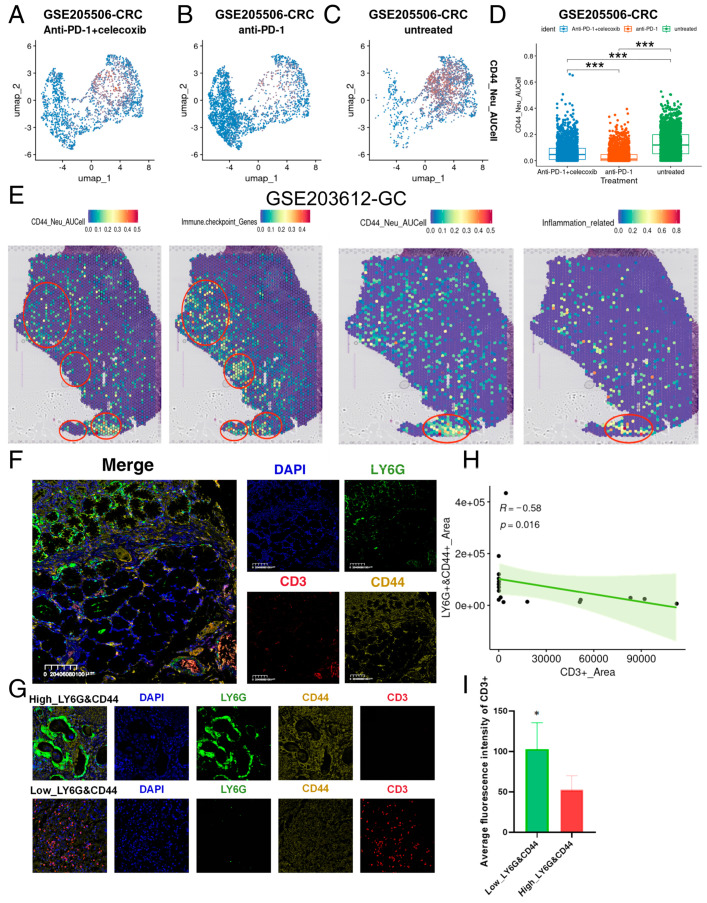
Validation of the correlation between CD44_NEU feature-related AUCell scores and immune efficacy. (**A**–**D**) UMAP plots of CD44_NEU feature-related AUCell scores characteristics in Anti-PD-1+celecoxib, anti-PD-1, and untreated groups in the GSE205506 dataset (orange represents high AUCell score; blue represents low AUCell score; the grouping is based on the average of CD44_NEU feature-related AUCell scores). CRC, colorectal cancer. (**E**) Distribution of CD44_NEU feature-related AUCell scores, immune checkpoint features AUCell scores, and ferroptosis features AUCell scores in the spatial transcriptome data of GIST1 GC in the GSE203612 dataset. (**F**) Immunofluorescence staining of *CD44*, *CD3*, and *LY6G* of tumor cells in patients with GC. (**G**) Immunofluorescence staining of *CD44*, *CD3*, and *LY6G* in different *CD44*+ and *LY6G*+ neutrophil GC patients. GC, gastric cancer. (**H**) The *CD3*+ region area and *CD44*+ and *LY6G*+ region area of each sample were measured using ImageJ (Fiji, version 1.54), and correlation analysis was performed. (**I**) Based on the median area of the *LY6G* and *CD44* double-positive region, GC tissue samples were divided into a high-positive region group and a low-positive region group. The average fluorescence intensity of the *CD3*-positive region was then compared between the different groups. * < 0.05; *** < 0.001.

To understand the relationship between CD44_NEU feature-related AUCell scores and immunotherapy and tumor immunotherapy efficacy, we extracted neutrophils from the GSE207422 dataset and calculated the CD44_NEU feature-related AUCell scores in the same way. We found that the CD44_NEU feature-related AUCell scores of neutrophils before treatment was significantly higher than that after treatment, and the infiltration of high-CD44_NEU feature-related AUCell-score neutrophils was significantly reduced after treatment (*p* < 0.05, Figure 5D,E). Similarly, NMPR patients with poorer post-treatment response had higher CD44_NEU feature-related AUCell scores and neutrophil infiltration with higher CD44_NEU feature-related AUCell scores compared to MPR patients (*p* < 0.05, Figure 5F,G). Then, we used the GSE205506 dataset and extracted neutrophils in the same way as before and validated the previous results. We found that compared with patients who had not been treated, patients treated with anti-PD-1 and anti-PD-1 combined with Celecoxib had significantly reduced CD44_NEU feature-related AUCell scores, and untreated patients had more neutrophil infiltration with a high CD44_NEU feature-related AUCell score (*p* < 0.05, Figure 6A–D). Subsequently, we used the GC spatial transcriptome data in GSE203612 to evaluate the CD44_NEU feature-related AUCell scores and the AUCell score of immune checkpoint features and inflammatory response features at all sites. We found that spatially, the CD44_NEU feature-related AUCell scores still had a certain correlation with the AUCell scores of immune checkpoint features and inflammatory response features (Figure 6E). To investigate the relationship between CD44_NEU and the infiltration of T cells, we performed multiple immunohistochemistry (mIHC) using a GC tissue microarray, with the details outlined in the Materials and Methods Section 4. The primary antibody was divided into three groups: *CD44*, *CD3* (a marker for T cells), and *LY6G* (a marker for neutrophils). We found that the *CD44* and *LY6G* fluorescence-double-positive region and *CD3* fluorescence-positive region are spatially repulsive, and the area of the *CD44* and *LY6G* fluorescence-double-positive region is significantly negatively correlated with the *CD3* fluorescence-positive region (Figure 6F–H). At the same time, we grouped the samples according to the area of *CD44* and *LY6G* fluorescence-double-positive region and counted the fluorescence intensity of *CD3*. It was found that the fluorescence intensity of *CD3* was significantly reduced in the samples with greater area in the *CD44* and *LY6G* fluorescence-double-positive region (Figure 6G–I).

**Figure 7:**
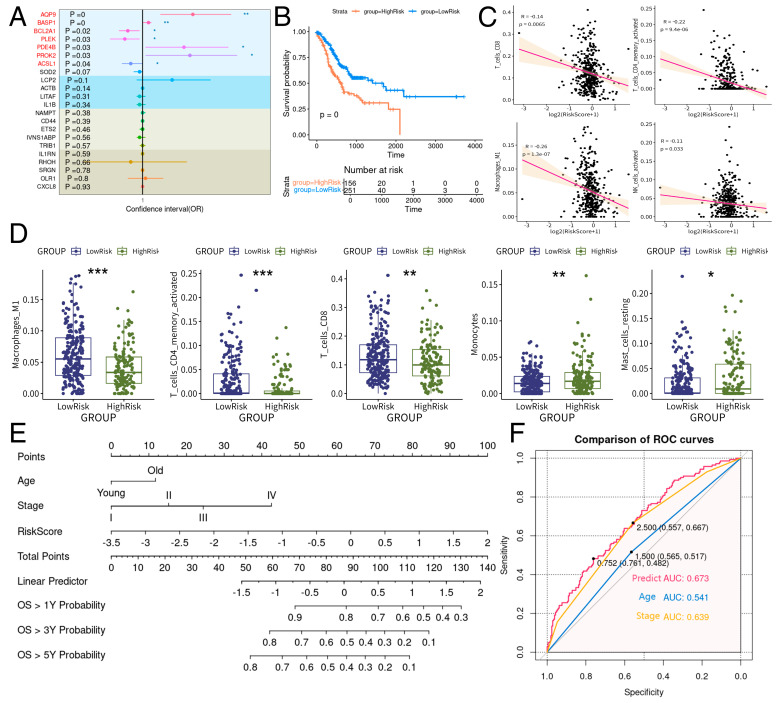
Establishment of a prognosis model based on the risk score of eight risk genes. (**A**) The forest map shows the results of COX regression for 22 key genes. (**B**) KM survival curve after constructing a multivariate COX regression model. The maximum selected rank statistic is used to determine the best truncation value for the conversion of continuous variable to binary variable to divide patients into high-risk and low-risk groups. (**C**) Correlation analysis of the predicted risk score with CD8 T cells, M1 macrophages, activated memory B cells, and activated NK cells predicted by CIBERSORTX in the TCGA-STAD dataset. (**D**) In the TCGA-STAD dataset, the maximum selected rank statistic is used to determine the best truncation value for the conversion of continuous variable to binary variable to divide patients into high-risk and low-risk groups, and the cell infiltration scores of M1 macrophages, resident dendritic cells, resident memory B cells, activated memory CD4 T cells, CD8 T cells, resident memory CD4 T cells, plasma cells, monocytes, and resident mast cells were compared using the CIBERSORTx prediction. (**E**) Prognosis prediction nomogram developed based on risk score, age, and pathological stage. (**F**) Validation ROC curve of the prognosis model. * < 0.05; ** < 0.01; *** < 0.001.

To understand the relationship between the core genes of CD44_NEU and prognosis, we first performed univariate COX regression on the core genes of CD44_NEU based on the TCGA-STAD transcriptome sequencing data and found that *AQP9*, *BASP1*, *BCL2A1*, *PLEK*, *PDE4B*, *PROK2*, and *ACSL1* were significantly related to survival (*p* < 0.05, Figure 7A). Subsequently, we used these seven risk genes for multivariate COX regression, obtained a risk model formula (= 0.12707264 × *AQP9* + 0.01384307 × *BASP1* − 0.04290294 × *BCL2A1* − 0.02679044 × *PLEK* + 0.10701552 × *PDE4B* + 0.06889772 × *PROK2* − 0.03419596 × *ACSL1*), and calculated the risk score. The maximum selected rank statistic was used to determine the best truncation value for the conversion of continuous variable to binary variable to divide patients into high-risk and low-risk groups, and a Kaplan–Meier (KM) survival curve was plotted (Figure 7B); patients in the high-risk group had significantly poor prognosis (*p* < 0.05). Subsequently, we used CIBERSORTX to assess the proportion of immune cell infiltration in each patient from the TCGA-STAD transcriptome sequencing data and then compared the immune infiltration differences between high- and low-risk groups based on risk grouping. We found that the risk score was significantly negatively correlated with the infiltration of CD8+ T cells, CD4+ memory T cells, M1 macrophages, and activated NK cells (Figure 7C and Figure S3A–K), and the infiltration of CD8+ T cells, CD4+ memory T cells, and M1 macrophages was significantly reduced in the high-risk group. Monocyte and mast cell infiltration increased significantly (*p* < 0.05, Figure 7D), suggesting a high correlation between risk score and immunosuppressive microenvironment formation. Then, we conducted multivariate COX regression combining clinical features and risk score and found that age, pathological stage, and risk score were significantly correlated with prognosis. Based on the multivariate COX results, we constructed a prognosis model and mapped a nomogram with RMS package (Figure 7E). The ROC curve was drawn for verification (Figure 7F), and the model predicted that the area of the ROC curve was 0.673. It was superior to age (ROC: 0.541) and pathological stage (ROC: 0.639). These results suggest that CD44_NEU-related key genes have partial prognostic ability, but other indicators need to be combined to further improve the prediction accuracy.

## 3. Discussion

Recent investigations have pinpointed neutrophils as a pivotal subgroup within the tumor microenvironment, with profound implications for tumor immunity [2]. The precision in predicting immune function and the characteristics of the tumor microenvironment is intimately tied to the response to immunotherapy. Consequently, elucidating the therapeutic mechanisms and identifying biomarkers that signify immunotherapy efficacy remain formidable challenges in this field [21,22].

In recent years, immunotherapy has become a new cancer treatment strategy. Recent reports have shown the construction of cancer prognosis models based on genes related to immune response, endoplasmic reticulum, and angiogenesis processes [23,24]. The predictability of immune function and the relationship between the tumor microenvironment and the response to immunotherapy are closely related, so how to determine the mechanism of efficacy and the biomarkers of the effect of immunotherapy is still a problem that needs to be solved in current immunotherapy.

In this study, we unraveled the intricate complexity and heterogeneity of tumor-associated neutrophils through a meticulous analysis of multiple datasets obtained from over 600,000 individual cells. Our investigation delved into the functional roles of these neutrophils, their impact on the immune system, and their modulation of the response to cancer therapy. Furthermore, we pinpointed the core genes that underpin these processes.

First, after undergoing strict quality control, our study found that in the tumor and metastatic stages, there are specific neutrophil subpopulations (CD44_NEU and CCL4_NEU) whose proportions increased significantly. This finding was consistently verified by multiple databases (such as GSE183904 and GSE163558), indicating the potential importance of these subpopulations in tumor progression. Functional enrichment analysis showed that CD44_NEU and CCL4_NEU subgroups were significantly enriched in several immune-related pathways, such as IL-17 signaling, NF-κB signaling, and toll-like receptor signaling. IL-17 is a highly versatile pro-inflammatory cytokine that is essential for host immune defense, tissue repair, the pathogenesis of inflammatory diseases, and cancer progression [25]. NF-κB is a family of transcription factors with classical and non-classical pathways, and its activation responds to various external stimuli involving inflammation, immune response, cell proliferation, differentiation and survival, and the development of immune cells [26]. IL-17 induces structural changes in the receptor, activating IκB kinase to phosphorylate and ubiquitinate the protein, leading to the release of NF-κB dimers P50 and P65 from the cytoplasm and their rapid translocation to the nucleus, thereby initiating NF-κB transcription. Studies have shown that IL-17A and IL-17RC interact to regulate the NF-κB/NOX1 pathway, affecting the progression of GC [27]. Therefore, intervening in the above pathways may be a potential way to improve the efficacy of clinical immunotherapy. The activation of toll-like receptors can reverse the immunosuppressive function of tumor-associated cells through metabolic regulation [28]. These results suggest that these neutrophil subpopulations may play an important role in immunotherapy and may become new therapeutic targets.

Building upon previous findings that CD44_NEU specifically proliferates during the tumor stage, we employed machine learning algorithms (Lasso, Univariate, RandomForest, and boruta) to discern the core gene set associated with CD44_NEU. Our analysis revealed that the core gene set comprises genes involved in inflammation and immunity (*CXCL8*, *IL1B*, *IL1RN*, *OLR1*, *PDE4B*, *PROK2*, and *SRGN*), tumor proliferation, invasion, and metastasis (*ACSL1*, *AQP9*, *BCL2A1*, *CD44*, *ETS2*, *RHOH*, and *TRIB1*) as well as energy metabolism and oxidative stress response (*LITAF*, *NAMPT*, and *SOD2*). Further exploration uncovered their intricate associations with diverse biological functions, encompassing angiogenesis, apoptosis, oxidative stress, and ferroptosis. Notably, in the high-CD44_NEU feature-related AUCell-scores group, a marked decrease in the immune checkpoint gene score was observed, reinforcing their potential contribution to immune mechanisms.

Hence, we delved deeper into the interplay between CD44_NEU feature-related AUCell scores and tumor immunity as well as its implications for tumor immunotherapy response. Leveraging the GSE207422 dataset, we uncovered a robust correlation between the AUCell score derived from the core gene set of CD44_NEU and both the efficacy of tumor immunotherapy and patient prognosis. Notably, neutrophils exhibiting high CD44_NEU feature-related AUCell scores underwent a significant decline posttreatment, while patients with suboptimal immunotherapy responses displayed heightened infiltration of these neutrophils. This underscores the potential of the CD44_NEU core gene set AUCell score as a potent predictor of immunotherapy response. This finding was independently validated in the GSE205506 dataset. Furthermore, multivariate Cox regression analysis illuminated the prognostic value of a risk score grounded in the CD44_NEU core gene, offering a novel perspective for personalized treatment strategies.

Ultimately, we devised a predictive model that integrates clinical characteristics with risk scores derived from TCGA-STAD transcriptome sequencing data, underscoring its promising application in GC patient management. Although the ROC value of this model was 0.673, it was still higher than the prediction ability of commonly used clinical pathological stage (ROC: 0.639) and age (ROC: 0.541) for prognosis, which proved that CD44_NEU did have an impact on the prognosis of patients. This model not only anticipates patient survival but also illuminates the intricate interplay between immune cell infiltration patterns and prognosis, thereby reinforcing our earlier discoveries. This comprehensive approach underscores the pivotal role of CD44_NEU in tumor progression and therapeutic response. At the same time, due to the limitations of retrospective studies, integrating more prognostic indicators in future studies can better improve the performance of prediction models and obtain more accurate risk cutoff values.

Previous studies have found that neutrophils regulate the immune response in the tumor microenvironment through the expression of various inflammation-related genes. In the core gene set of CD44_NEU, we found that for some genes, such as *CXCL8* (*IL-8*), the main role is to stimulate the migration and invasion of tumor cells, which can help tumor cells escape [29]. *IL-1β* and *IL1RN* can promote tumor angiogenesis and the recruitment of immune suppressor cells. Overexpression of *OLR1* is involved in enhancing the migration of tumor cells through *NF-κB* activation [30]. *SRGN* plays a key role in the interaction between tumor and stroma and reprogramming to an invasive and immunosuppressive tumor microenvironment in *TTF-1* negative LUAD [31]. *PDE4B* promotes the development of tumors by regulating the level of intracellular cAMP, affecting the production of inflammatory mediators and the activation state of immune cells [32]. *PKR2* is a ligand of a G protein-coupled receptor involved in regulating tumor growth, angiogenesis, and metastasis in the tumor microenvironment [33].

CD44_NEU also expresses a series of genes related to tumor proliferation, invasion, and metastasis. *ACSL1* promotes cell proliferation and migration, and inhibiting *ACSL1* significantly inhibits the growth of prostate xenograft tumors in the body [34]. *BCL2A1* is a highly regulated nuclear factor κB (*NF-κB)* target gene that is overexpressed in various tumors [35]. *CD44*, *ETS2*, *RHOH*, and *TRIB1* also affect the proliferation and invasion ability of tumor cells by regulating the expression of downstream genes [36,37,38].

In addition, marker genes that regulate energy metabolism and oxidative stress response in the tumor microenvironment are also involved in tumor development. *LITAF* is a downstream target of *AMPK* that inhibits cancer cell growth by increasing the expression of *TNFSF15* and inhibiting angiogenesis [39]. NAMPT can inhibit T-cell-mediated anti-tumor immunity through enzyme-independent functions. SOD2 is a tumor suppressor protein whose main function is to eliminate superoxides in cells. After being acetylated by K68, SOD2 increases the level of H_2_O_2_ in the mitochondria, thereby promoting the invasion and metastasis of tumors [40,41].

In summary, our study provides new insights into the remodeling mechanism of the TME in GC and the mechanism of immune response to tumors, especially the important role played by neutrophils in this process. These findings not only enrich our understanding of the tumor immune microenvironment but also provide important scientific basis for the development of new diagnostic and treatment strategies.

However, there are some limitations in this study. First, although we found that CD44_NEU can be used as a marker for the response to immunotherapy, and the CD44_NEU feature-related AUCell score is related to the response to treatment, the cutoff value of the CD44_NEU feature-related AUCell scores still needs to be further confirmed by large-scale scRNA-seq data. Second, our study used datasets from multiple cancers, and some patients received combined treatment of immunotherapy and chemotherapy, which may affect our interpretation of the response to immunotherapy. At the same time, different ethnic and regional sample sources in different databases may also introduce potential confounding factors. Finally, the results of our study need to be further verified by basic research and prospective studies. Evaluating the effect of neutrophils on the cancer immune microenvironment in real-world settings through clinical studies with larger sample sizes could strengthen the credibility and applicability of this model to a broader patient population.

## 4. Materials and Methods

### 4.1. Single-Cell Dataset and RNA-Seq Dataset Collection

To gather transcriptomic and clinical data for patients, the researchers accessed two databases: the Cancer Genome Atlas (TCGA) (https://www.cancer.gov/, accessed on 1 October 2024) and the Gene Expression Omnibus (GEO) (https://www.ncbi.nlm.nih.gov/geo/, accessed on 1 October 2024) [42]. The TCGA-STAD dataset comprised 437 samples from GC patients. Genomic analysis (FPKM) and clinical data were collected from patients up to 8 May 2022. We converted the TCGA-FPKM STAD data into transcripts per million (TPM) for comparison. The GEO data came from five sources: the GSE163558 dataset (10 samples, GC) [43], the GSE183904 dataset (40 samples, GC) [44], the GSE205506 dataset (40 samples, colorectal cancer) [45], the GSE207422 dataset (39 samples, lung cancer) [46], and the GSE203612 dataset (GC, spatial transcriptomics) [47].

### 4.2. Raw Data Processing and Quality Control

The single-cell sequencing dataset in this study was studied using a uniform original processing method and normalization process to obtain a unique molecular identifier (UMI) matrix for each sample. Normalization was performed using the Seurat package (version 3.2.2) [48] in R. All normalized data were log2-transformed. Three quality control measures were adopted, with exclusion criteria as follows: (1) <200 expressed genes or >2500 expressed genes, (2) mitochondrial genes >20% of UMIs, and (3) nCount_RNA > 10,000 UMIs. The Harmony algorithm was executed to eliminate batch effects [49].

### 4.3. Data Integration, Unsupervised Clustering, and Cell Type Annotation

Principal component analysis (PCA) was performed on the integrated expression matrix using the RunPCA function, and the first 20 principal components (PCs) were used in the FindNeighbors function. The resolution parameter of the FindClusters function varied for different cell types, with the first execution at 0.20 and subsequent executions at 0.5. The RunUMAP function with the same PCs and other default parameters was used to perform uniform manifold approximation and projection (UMAP) for two-dimensional visualization. The main cell lineages were assigned to each cell cluster based on the abundance of classic marker genes, and differentially expressed genes (DEGs) for each cluster were identified using the FindAllMarkers function with parameters “min.pct = 0.3, only.pos=T”. The DEGs were subjected to GO and KEGG functional annotation and enrichment analysis using the clusterProfiler software package (version 2.11) [50], with GO and KEGG enrichment annotations with an false-discovery rate (FDR) < 0.05. Deconvolution assessment of immune infiltration of TCGA-STAD was performed using the immune infiltration reference document provided by CIBERSORTx (https://cibersortx.stanford.edu/, accessed on 1 September 2024) [51].

### 4.4. Machine Learning of Key Gene Features

The counts matrix from scRNA data was input with specific gene markers, and the GeneSelectR package (https://github.com/dzhakparov/GeneSelectR) was used to identify genes related to the CD44_NEU subpopulation through Lasso, Univariate, RandomForest, and boruta, four machine learning algorithms. The intersection of the results of the four algorithms was taken as the key genes.

### 4.5. Gene Set AUCell Score

The AUCell (version 1.14.0) [52] package was used to evaluate the enrichment score of specific gene sets in scRNA data.

### 4.6. Establishment of Nomograms and Independent Prognosis Analysis

Considering clinical variables and survival outcomes, univariate and multivariate COX analyses were used to evaluate the independence of the risk score. Kaplan–Meier survival curves were plotted using the survminer (version 0.4.8) and survival (version 3.5.5) R packages, including cumulative event tables and log-rank tests. The “survivalROC” script was used to evaluate the area under the curve (AUC) of the prognosis model.

### 4.7. Multiplex Immunohistochemistry

To investigate the expression intensity and spatial distribution of *CD44*, *LY6G* (a marker for neutrophils), and *CD3* (a marker for T cells) in tumor tissues, we conducted multiplex immunohistochemistry (mIHC) using the PANO Multiplex IHC Kit (10234100100; Panovue, Beijing, China), following the manufacturer’s instructions. A separate GC tissue microarrays (TMAs) were purchased and used in this study (YEPCOME Biotechnology Co., Shanghai, China). The TMA slides consisted of 30 GC tissues and 30 noncancerous tissues. All 60 cores with a 1.5 mm diameter were arranged in paraffin blocks in TMAs. Detailed clinicopathological features such as gender, age, tumor grades, depth of tumor invasion (T), lymph node metastasis (N), and distant metastasis (M) are given in Supplementary Material S4. Briefly, GC and paracancerous tissue chips were incubated in a hot-air oven at 65 °C overnight. Subsequently, they were deparaffinized in fresh xylene for 10 min three times, rehydrated through a graded series of ethanol (100% to 95% to 70%), and then washed three times with PBS. Antigen retrieval was performed using microwave heating, followed by cooling in an ice water bath for at least 15 min. After blocking in blocking solution (0018001120; Panovue, Beijing, China) for 15 min at room temperature, the TMA sections were incubated with the primary antibody for 30 min, followed by the secondary antibody for 10 min, and then stained with TSA Opal fluorophores for 10 min. These steps (antigen retrieval, blocking, primary antibody incubation, and TSA Opal staining) were repeated for each marker. Finally, the TMA sections were counterstained with DAPI (D9542; Sigma-Aldrich, St. Louis, MO, USA) for 5 min and mounted. Whole TMA section imaging was conducted using the panoVIEW VS200 imaging system (Panovue, Beijing, China). The primary antibodies used were *CD44* (ab254530; 1:200 dilution; Abcam, Cambridge, UK), *LY6G* (ab238132; 1:300 dilution; Abcam, Cambridge, UK), and *CD3* (ab16669; 1:500 dilution; Abcam, Cambridge, UK). Multiple immunofluorescence images were analyzed using ImageJ (Fiji, version 1.54) [53]. Specifically, we first employed the image processing software ImageJ to accurately measure the area of *LY6G* and *CD44* double-positive regions and *CD3*-positive regions in each GC tissue sample. Subsequently, we utilized the ggplot2 and ggpubr packages in R to conduct correlation statistical analysis on these data and generate corresponding visualization charts. To further explore the relationship between *LY6G* and *CD44* double-positive regions and *CD3*-positive regions, we divided the GC tissue samples into a high-positive region group and a low-positive region group based on the median area of *LY6G* and *CD44* double-positive regions. Then, we statistically compared the average fluorescence intensity of *CD3*-positive regions between the two groups to reveal the differences between them.

### 4.8. Statistical Analysis

All data analyses were conducted using the R platform, specifically version 4.4.3. To compare continuous variables between two subgroups, we applied either Student’s *t*-test or the Wilcoxon rank-sum test. Differences among three groups were assessed using either one-way ANOVA or the Kruskal–Wallis test. For evaluating the correlation between normally distributed variables, Pearson correlation was employed, whereas Spearman correlation was used for non-normally distributed variables. To control for the inflation of type I errors in multiple testing scenarios, the Benjamini and Hochberg (BH) method was utilized to estimate the FDR, which adjusts the *p*-values to account for the number of tests conducted. For survival analysis, the “survminer” R package was leveraged to perform Kaplan–Meier analysis and log-rank tests, allowing us to assess survival disparities between groups. All statistical image visualizations were created using the R packages ggplot2 and ggpubr. Notably, all analyses were conducted using the default parameters provided by the respective functions and packages, unless otherwise specified. The implementation of the BH method for FDR control ensured that our results were more robust and reliable, minimizing the likelihood of false positives arising from multiple comparisons.

## 5. Conclusions

Utilizing both scRNA-seq and bulk RNA-seq methodologies, we elucidated the distinct profiles of specific neutrophil subpopulations and uncovered differences in their characteristics between patients demonstrating favorable versus unfavorable responses to immunotherapy. Notably, we identified the CD44_NEU feature-related AUCell score as a potential biomarker predictive of immunotherapy outcomes. Furthermore, we developed a predictive model anchored on *AQP9*, *BASP1*, *BCL2A1*, *PLEK*, *PDE4B*, *PROK2*, and *ACSL1* genes, which effectively categorizes the CD44_NEU subgroup and exhibits prognostic prediction capabilities, thereby presenting a valuable tool for clinical prediction.

## Data Availability

This study is based on sequencing datasets from previously published research. The datasets supporting the conclusions of this article are available in the (GEO) database and the cancer genome atlas (TCGA) database. The URL for the database used can be found below: TCGA-STAD: https://www.cancer.gov/ (accessed on 3 June 2024); GSE163558: https://www.ncbi.nlm.nih.gov/geo/query/acc.cgi?acc=GSE163558 (accessed on 3 June 2024); GSE183904: https://www.ncbi.nlm.nih.gov/geo/query/acc.cgi?acc=GSE183904 (accessed on 3 June 2024); GSE205506: https://www.ncbi.nlm.nih.gov/geo/query/acc.cgi?acc=GSE205506 (accessed on 3 June 2024); GSE207422: https://www.ncbi.nlm.nih.gov/geo/query/acc.cgi?acc=GSE207422 (accessed on 3 June 2024); GSE203612: https://www.ncbi.nlm.nih.gov/geo/query/acc.cgi?acc=GSE203612 (accessed on 3 June 2024).

## Supplementary Materials

The supporting information can be downloaded at https://www.mdpi.com/article/10.3390/ijms252312715/s1.
