# Supplementary material for: Machine Learning Integration with Single-Cell Transcriptome Sequencing Datasets Reveals the Impact of Tumor-Associated Neutrophils on the Immune Microenvironment and Immunotherapy Outcomes in Gastric Cancer
Source: Int J Mol Sci. 2024 Nov 26;25(23):12715. doi: 10.3390/ijms252312715 (PMC11641192; doi:10.3390/ijms252312715)
Supplement: Supplementary file 1 [file ijms-25-12715-s001.zip › Supplementary picture.pdf]

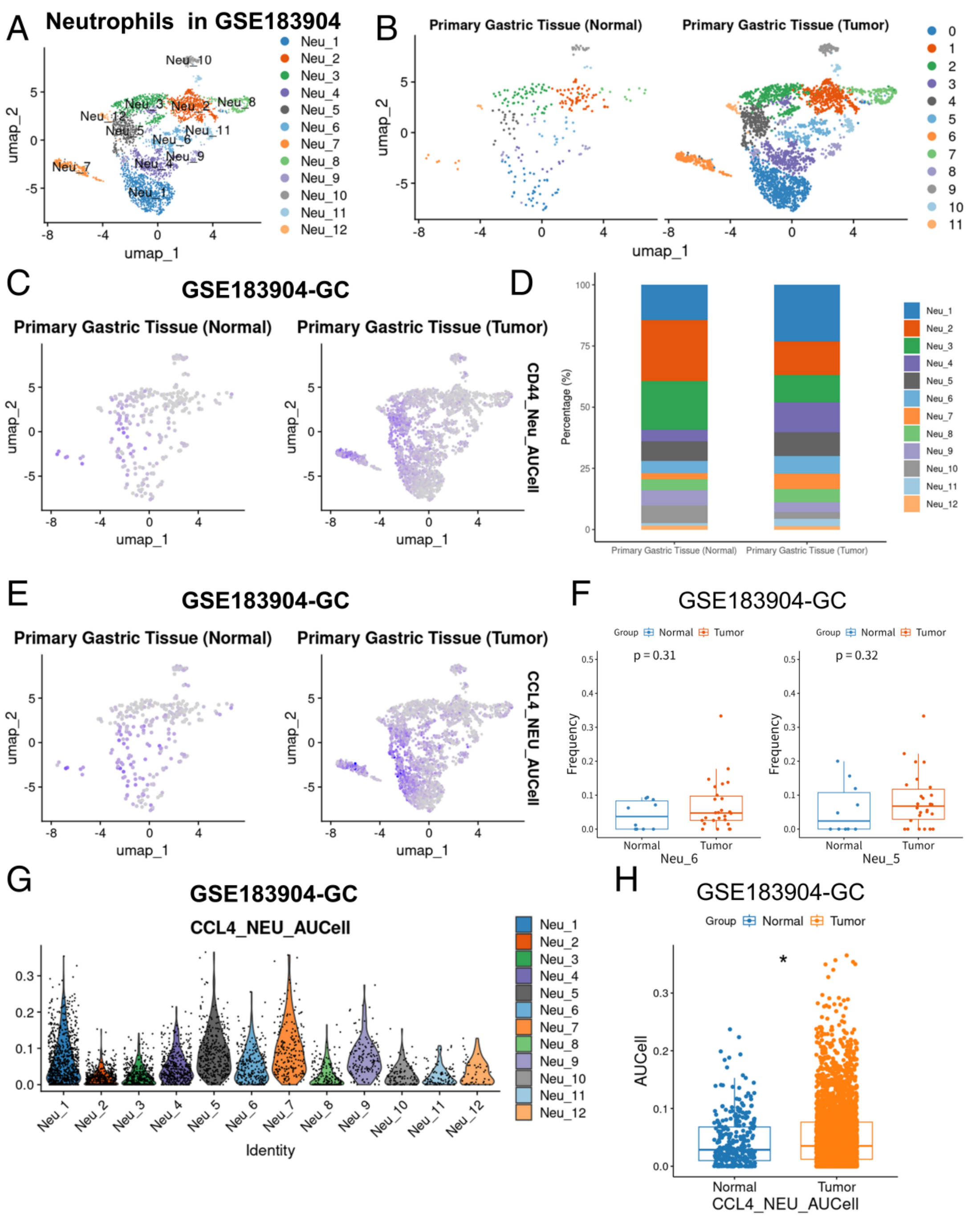

Figure S1. **A**, UMAP of neutrophil dimension reduction clustering in GSE183904 gastric cancer single cell sequencing database. **B**, Contrast UMAP of neutrophils in adjacent normal tissue and gastric cancer tissue. **C**, Comparison of CD44_NEU feature-related AUCell scores scores in adjacent normal tissues and gastric cancer tissues. **D,** Histogram of comparison of neutrophil abundance in adjacent normal tissue and gastric cancer tissue. **E,** Comparison of CCL4_NEU feature-related AUCell scores scores in adjacent normal tissues and gastric cancer tissues. **F,** statistical test diagram of Neu_6 and Neu_5 abundance in adjacent normal tissue and gastric cancer tissue. **G,** CCL4_NEU feature-related AUCell scores scores of neutrophil subsets

compared with violin chart. **H,** statistical test diagram of neutrophil CCL4_NEU feature-related AUCell scores scores in paracancerous normal tissue and gastric cancer tissue.

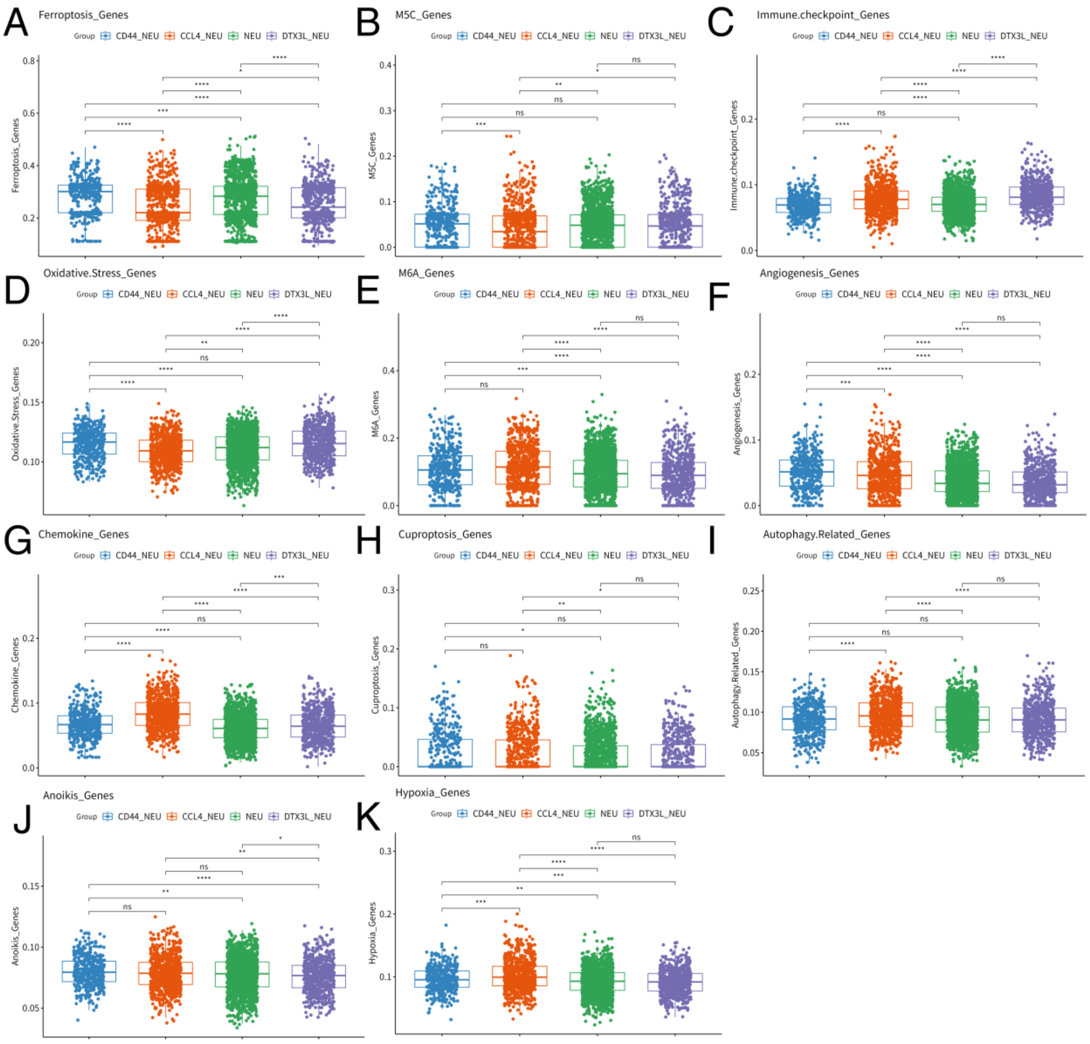

Figure S2. A-K, comparison of 11 biological functional characteristics of different neutrophil subsets from the GSE163558 gastric cancer single-cell sequencing database.* < 0.05, ** < 0.01, *** < 0.001, **** < 0.0001, ns: Not significant.

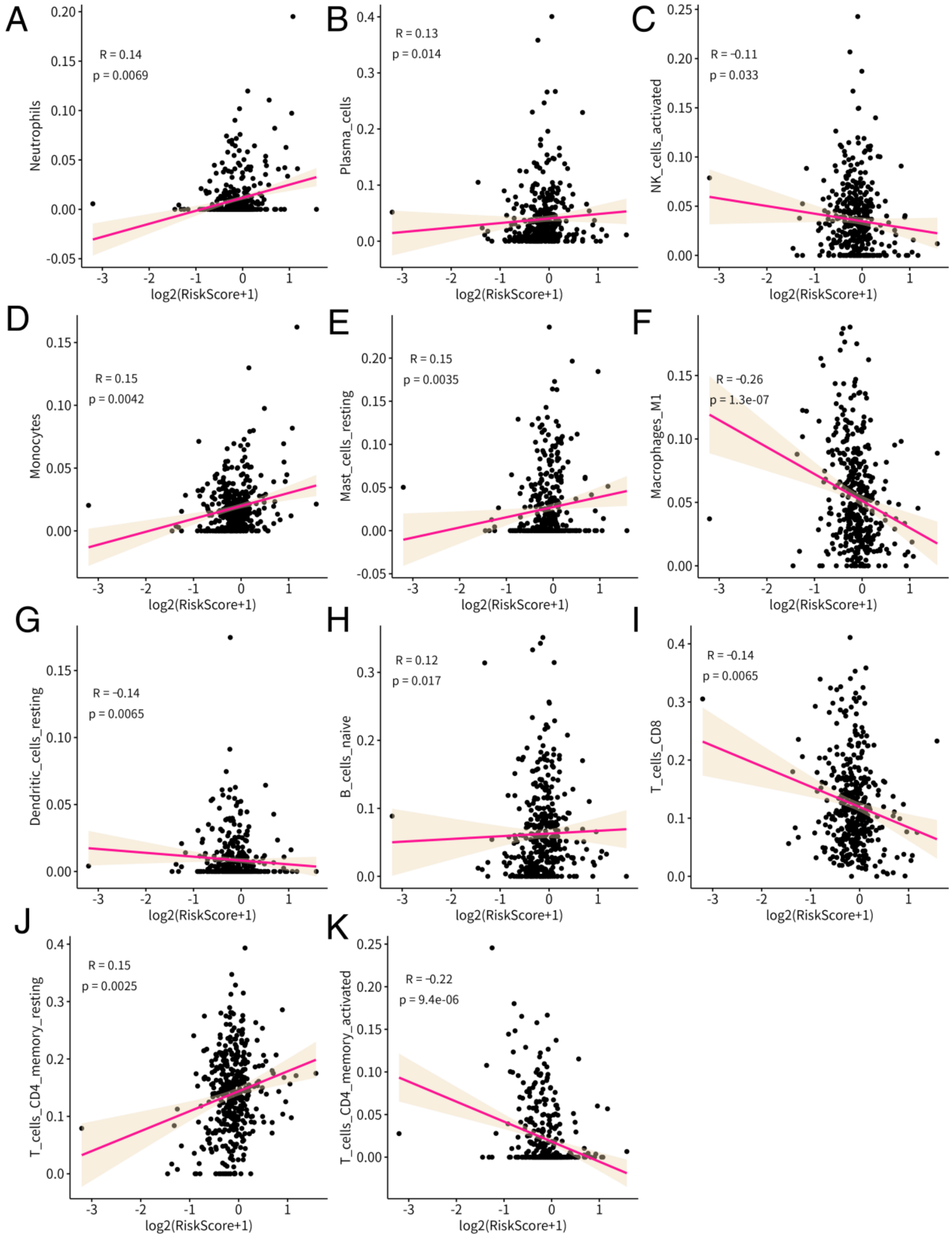

Figure S3. A-K, the transcriptome sequencing data of gastric cancer samples from TCGA-STAD were used to calculate the risk score according to the established prognostic model, and then the correlation between the risk score and the content of 11 kinds of immune cells was analyzed. Immune cell content data were obtained from deconvolution results of CibersortX.
